# Common and Unique Respiratory Health Risk Induced by Urban-Rural PM_2.5_ in the Chengdu-Chongqing Economic Circle

**DOI:** 10.3390/toxics14060531

**Published:** 2026-06-20

**Authors:** Xuan Li, Zhipeng Wang, Yuhan Feng, Mi Tian, Shike Shang, Yang Chen, Jingli Qian, Shumin Zhang, Yulan Yang

**Affiliations:** 1 Institute of Basic Medicine, North Sichuan Medical College, Nanchong 637000, China; llxuan121@163.com (X.L.);; 2 School of Public Health, North Sichuan Medical College, Nanchong 637000, China; 3 Key Laboratory of Three Gorges Reservoir Region’s Eco-Environment, Ministry of Education, Chongqing University, Chongqing 400045, China; 4 Department of Traditional Chinese Medicine and Rehabilitation, People’s Hospital of Chongqing Liang Jiang New Area, Chongqing 401121, China; 5 Research Center for Atmospheric Environment, Chongqing Institute of Green and Intelligent Technology, Chinese Academy of Sciences, Chongqing 400714, China

**Keywords:** PM_2.5_, respiratory system, health risk, KIF20A, Chengdu-Chongqing economic circle, urban-rural

## Abstract

Fine particulate matter with a diameter ≤2.5 μm (PM_2.5_) pollution poses a global public health crisis, demonstrating significant threats to human health. This study focused on the strategically important Chengdu-Chongqing Economic Circle in western China, systematically comparing the toxic effects of urban and rural PM_2.5_ across five levels. PMF and regression analysis were used to identify source contributions, dual-omics to pinpoint key molecules, and epidemiological data with a GAM model to assess health risks. Findings demonstrate that rural PM_2.5_ possesses greater biotoxicity than its urban counterpart. Cytotoxicity in urban and rural PM_2.5_ originated from road dust/vehicle emissions and biomass burning, respectively. Subsequently, integrated omics and molecular biology analyses identify kinesin family member 20A (KIF20A) as a shared key target, which mediates toxicity induced by both urban and rural PM_2.5_. Finally, epidemiological analysis reveals that females and ≥65 years old exhibit relatively high sensitivity to urban PM_2.5_ exposure trends, with rhinitis showing a comparatively higher impact among various related diseases. The novelty of this work lies in its pioneering application of a multi-tiered investigative approach. This approach spans “environmental samples-cellular mechanisms-population health” within the Chengdu-Chongqing economic circle context, systematically elucidating common and distinct respiratory health risk of urban and rural PM_2.5_. This work offers a vital scientific foundation for advancing region-specific, precise air pollution prevention and control measures.

## 1. Introduction

Fine particulate matter (PM_2.5_), a critical global health risk, induces severe multiorgan toxicity. The 2023 Lancet Countdown on Health and Climate Change attributed approximately 100 deaths per 780,000 population to PM_2.5_ exposure [1]. The respiratory system serves as the primary target organ. It develops oxidative stress, inflammation, and immune dysregulation upon exposure. These effects exacerbate asthma and chronic obstructive pulmonary disease (COPD) and increase lung cancer risk [2,3,4]. Thus, PM_2.5_-related respiratory diseases represent a substantial public health burden.

Epidemiological studies have conclusively demonstrated the adverse respiratory health effects of urban PM_2.5_ at a global scale [5]. Specifically, each 10 µg/m^3^ increase in urban PM_2.5_ concentration is associated with significant health impacts. These include 2.07%, 0.58%, and 8.00% increases in disease prevalence, mortality, and hospitalization rates, respectively [6,7,8]. In contrast, research on PM_2.5_-related health impacts in rural areas remains notably scarce and fragmented worldwide. Limited available evidence indicates a stronger association between 7-day cumulative PM_2.5_ exposure and COPD mortality in rural versus urban settings [9]. This pattern not only suggests potential urban-rural differences in PM_2.5_ toxicity but also highlights significant disparities in respiratory disease risk distribution.

PM_2.5_ sources show clear geographic differences [10]. Urban PM_2.5_ mainly originates from heavy traffic and industry, while rural PM_2.5_ is more influenced by farming and biomass burning. Its chemical composition is diverse, containing water-soluble ions, carbon compounds, and metals, etc. These differences lead to varying health impacts across regions [11]. Research has begun to uncover how urban and rural PM_2.5_ cause harm. However, knowledge gaps remain. Urban PM_2.5_’s effects are better understood, with several disease-related pathways identified (NOX4/Nrf2, TLR2/TLR4/MyD88, IL-13/IL-17A) [12,13,14]. Rural PM_2.5_ research is less advanced, with only one pathway (RRM2/CCND1) found to affect cell growth and death [15].

Under the combined influence of complex basin topography, high population density, and growing energy consumption, the Chengdu-Chongqing economic circle—a national-level development zone—faces a dual atmospheric pollution challenge characterized by the coexistence of photochemical smog and PM_2.5_. As the economic engine of Southwest China, this region boasts the densest population, the most robust industrial base, and the highest degree of openness, centered around the Chengdu-Chongqing megacity cluster [16]. Though previous studies have preliminarily elucidated the compositional characteristics, source apportionment, and pollution patterns of PM_2.5_ in this area [17,18], the differences in PM_2.5_-related health risks between urban and rural areas within the economic circle remain poorly understood.

Current research lacks systematic investigations into the urban-rural disparities of PM_2.5_ spanning source, toxicological effects, pathogenic mechanisms and epidemiology. Therefore, this study employs an interdisciplinary approach to systematically compare the health risks of urban- and rural-PM_2.5_, with a focus on the Chengdu-Chongqing economic circle. Through multi-scale and multi-level analyses, we aim to elucidate the intrinsic causes of urban and rural-PM_2.5_ health risk disparities and their differential pathogenic mechanisms, thereby providing scientific evidence for region-specific health risk assessment and precision prevention strategies.

## 2. Materials and Methods

### 2.1. Collection and Extraction of PM_2.5_

Urban and rural PM_2.5_ sampling sites in Shuitu Subdistrict, Liangjiang New Area, Chongqing (106.54° E, 29.81° N) and Guihong Village, Guanghan City, Sichuan Province (104°21′ E, 30°99′ N). The urban sampling site was situated in a residential zone with no proximate heavy industrial activities, whereas the rural site was surrounded by rice paddies and exhibited no high-rise structures or industrial enterprises within a 5 km radius. The two sampling locations were geographically separated by 260 km. Sampling was conducted from October 2019 to February 2022 during winter period using a high-volume air sampler (Thermo Fisher Scientific, Waltham, MA, USA) to capture atmospheric PM_,,_ onto quartz fiber filters (Hangzhou Whatman Filtration Paper Co., Ltd., Hangzhou, China, 18 cm × 23 cm). The sampling flow rate was maintained at 1.05 m^3^/min with a daily collection period of 23 h.

A 3 cm^2^ section of each filter was ultrasonically extracted with methanol, dried by nitrogen gas, and reconstituted in DMSO to prepare for a 200 mg/mL stock solution, which was stored at −20 °C. Finally, the stock solution was serially diluted to gradient concentrations for toxicological assessments.

### 2.2. Source Analysis of PM_2.5_

Organic carbon (OC) and elemental carbon (EC) were quantified using a DRI Model 2015 carbon analyzer (Magee Scientific, Berkeley, CA, USA). OC was measured under oxygen-free conditions (580 °C), while EC was determined through MnO_2_-catalyzed oxidation in 2% O_2_ (840 °C), with both converted to CO_2_ and detected by NDIR. Water-soluble ions (Na^+^, NH_4_^+^, K^+^, Mg^2+^, Ca^2+^, F^−^, Cl^−^, SO_4_^2−^, NO_3_^−^) were analyzed by ion chromatography (Thermo Fisher, USA) after column separation and blank correction [19]. The metal composition of PM_2.5_ was determined using energy-dispersive X-ray fluorescence spectroscopy (Epsilon4, Almelo, The Netherlands). Quality control was ensured using NIST SRM 2783 standard reference material [20]. The PMF 5.0 model developed by the U.S. EPA was employed to analyze the chemical composition data of PM_2.5_ samples [21]. Input parameters included concentration datasets with corresponding uncertainties for all chemical components. Through iterative computation, the optimal solution was obtained to derive source contribution profiles.

### 2.3. CCK-8

Using the CCK-8 assay, we evaluated the cell viability of two immortalized cell lines (A549, type II alveolar epithelial cells, and BEAS-2B, bronchial epithelial cells). Both were obtained from Wuhan Procell Life Science & Technology Co., Ltd. (Wuhan, China). Adults inhale approximately 10 to 11.5 cubic meters of air per day; under physical activity or stress, the respiratory rate can increase to 20 cubic meters. The average liquid volume of the human airways and lungs is 12.4 milliliters. To simulate real-world exposure scenarios under extreme pollution conditions, and based on data from the National Environmental Monitoring Center (where the maximum PM_2.5_ concentrations in Chongqing and North China can reach 350 and 450 μg/m^3^, respectively), the maximum daily exposure concentration for adults can reach 550 and 725 μg/mL, respectively (calculated as 350 × 20/12.4 and 450 × 20/12.4) [22]. Therefore, this study set the maximum exposure concentration of PM_2.5_ to 700 μg/mL.

Both cell lines were seeded in 96-well plates and cultured at 37 °C with 5% CO_2_ for 12 h. The cells were then treated with urban and rural PM_2.5_ at concentrations of 0, 100, 300, 500, and 700 μg/mL for 24 and 48 h. Subsequently, 10 μL of CCK-8 reagent (Coolaber Technology, Beijing, China) was added to each well and incubated at 37 °C for 1 h. The absorbance was measured at 450 nm using a multifunctional microplate reader (Thermo Fisher). Cell viability was calculated as: [A(PM_2.5_) − A(blank)]/[A(control) − A(blank)] × 100%. A(blank): CCK-8 + medium; A(control): CCK-8 + medium + cells; A(PM_2.5_): CCK-8 + PM_2.5_ + medium + cells.

### 2.4. Contribution Analysis of Urban and Rural PM_2.5_ Sources and Their Toxic Effects

Multiple linear regression analysis was performed separately for urban and rural samples (N = 10 per group), with cell viability (CCK-8 assay) as the dependent variable and the pollution sources identified by PMF as independent variables. A stepwise backward elimination method was employed for variable selection. The adjusted R^2^ values of all final models exceeded 0.8, and the variance inflation factor (VIF) for all independent variables was below 5, indicating no serious multicollinearity. The contribution of each statistically significant pollution source (*p* < 0.05) was determined by normalizing the product of the absolute value of the standardized partial regression coefficient (β) and the standard deviation of the corresponding independent variable.

### 2.5. LDH

Cell culture conditions, PM_2.5_ exposure gradients, and durations were consistent with the CCK-8 assay. The procedure followed the LDH assay kit instructions (Nanjing Jiancheng Bioengineering Institute, Nanjing, China). Sodium pyruvate standard solution, test samples, substrate buffer, 2,4-dinitrophenylhydrazine, and NaOH were sequentially added. Absorbance at 440 nm (A) was measured using a microplate reader (Synergy Biotechnology, Shanghai, China). LDH activity (U/L) = [A(test) − A(control)]/[A(standard) − A(blank)] × C(standard) × N × 1000, where N represents the dilution factor.

### 2.6. Transmission Electron Microscopy (TEM)

Following 24 h exposure to urban (600 μg/mL) or rural (500 μg/mL) PM_2.5_ (IC50 concentrations), A549 and BEAS-2B cells were processed for TEM analysis. The samples underwent glutaraldehyde fixation, ethanol dehydration, epoxy resin embedding, thermal polymerization, ultrathin sectioning (70 nm), and uranyl acetate/lead citrate staining. Ultrastructural examination was conducted using a JEOL TEM (Tokyo, Japan) operating at 80 kV, with systematic image acquisition.

### 2.7. Fourier Transform Infrared Spectroscopy (FTIR)

Cell culture and PM_2.5_ exposure conditions matched the TEM experiment. After 24 h exposure, cells were fixed with 75% ethanol (1 mL) for ≥30 min, centrifuged at 1500 for 5 min and resuspended in 20 μL ethanol. A 10 μL aliquot was dried on an FTIR window (Thermo Fisher, USA). Spectra were acquired after background subtraction. Data were smoothed and normalized using OMNIC 9.7 and Origin 2021 software.

### 2.8. Apoptosis

Apoptosis was analyzed by flow cytometry. Cells were seeded in 6-well plates for 12 h. Subsequently, they were exposed to urban (600 μg/mL) or rural (500 μg/mL) PM_2.5_ (IC50) for 24 h. Cells were then digested with EDTA-free trypsin, centrifuged at 1500× *g* for 5 min, and washed twice with cold PBS (4 °C). Following the apoptosis kit protocol (Elabscience, Wuhan, China), samples were analyzed using a flow cytometer (Agilent, Santa Clara, CA, USA). Data were processed by FlowJo 10.8.1 software.

### 2.9. Cell Cycle

Pretreatment conditions were identical to those in the apoptosis experiment. After exposure, cells were collected and resuspended in PBS (Phosphate-Buffered Saline) to adjust the cell concentration to 1 × 10^6^ cells/mL. Cells were then centrifugated at 1500× *g* for 5 min. The supernatant was discarded, and 500 μL of pre-cooled 70% ethanol was added. Cells were fixed overnight at 4 °C and subsequently washed with PBS. According to the cell cycle detection kit instructions (Solarbio, Beijing, China), RNase A solution and PI staining solution were sequentially added. Finally, cells were detected using a flow cytometer (Agilent Flow Cytometer, Santa Clara, CA, USA), and the cell cycle distribution was analyzed using ModFit LT 5.0 software.

### 2.10. Transcriptome and Proteome Sequencing

A549 cells were seeded in 6 cm dishes and cultured for 12 h. Cells were then exposed to urban PM_2.5_ at 600 μg/mL and rural PM_2.5_ at 500 μg/mL (IC50) for 24 h. Total RNA was extracted using the TRIzol method and sent to Novogene Bioinformatics Technology Co., Ltd. (Beijing, China). for transcriptome sequencing. For each sample, 0.2 μg of RNA was used to construct sequencing libraries with the NEBNext^®^ Ultra™ RNA Library Prep Kit for Illumina^®^ (NEB, Ipswich, MA, USA). Cluster generation was performed on the cBot cluster generation system using the TruSeq PE Cluster Kit v3-cBot-HS (Illumina, San Diego, CA, USA). Sequencing was ultimately conducted on the Illumina NovaSeq platform [15,23].

Preliminary processing for proteomics sequencing followed the same protocol as transcriptomics. Cell samples were flash-frozen in liquid nitrogen and subsequently sent to Jingjie Biotechnology for proteomic sequencing. Separation was performed using a NanoElute ultra-high performance liquid chromatography system, followed by ionization via a capillary ion source. Raw data were acquired on a timsTOF Pro 2 mass spectrometer. The resulting mass spectrometry data were analyzed with MaxQuant v.1.6.15.0. [15,23]. RNA-seq data are accessible at the GEO database under accession number GSE246730. Proteomics data are available via the ProteomeXchange database with accession number PXD047163.

### 2.11. Analysis of Key Molecules (Genes/Proteins), GO Terms, KEGG Pathways, and Disease Associations

Differentially expressed genes (DEGs) were identified using the criteria of adjusted *p*-value (Padj) < 0.05 and |log2FC| > 1.0. Differentially expressed proteins (DEPs) were selected with *p*-value < 0.05 and expression ratio >1.5 or <1/1.5. Subsequently, a protein–protein interaction (PPI) network was constructed using the STRING database (http://string-db.org) with a confidence score threshold of ≥0.7. Based on the PPI network, the top 50 DEGs/DEPs from each algorithm in CytoHubba were identified. Their intersection from 12 algorithms was determined. The top 50 molecules ranked by intersection frequency were finally defined as the key molecular set.

For transcriptomics and proteomics, Gene Ontology (GO) terms and Kyoto Encyclopedia of Genes and Genomes (KEGG) pathway enrichment (*p* < 0.05) of the key molecular set were performed on the Novogene Cloud (clusterProfiler v3.0.3) and PTM BioLab Cloud, respectively. KEGG analysis filtered out non-mechanistic pathways, including those for human diseases and organ systems. Respiratory disease-associated molecules (genes/proteins) were identified via MalaCards (ranked by Search Score). GO terms and KEGG pathways linked to respiratory diseases were analyzed using CTDbase (ranked by Inference Score).

### 2.12. Western Blot and Transfection Experiments

Exposure conditions matched omics experiments. Primary antibodies included KIF20A (66590-1-Ig, Proteintech, Wuhan, China) and β-actin (AB0035, Abways Shanghai, China). Secondary antibodies included goat anti-mouse (SA00001-1, Proteintech) and goat anti-rabbit (TA373083, Origene, Beijing, China). KIF20A overexpression lentivirus and control virus were provided by VectorBuilder (Guangzhou, China). Procedures followed references [15,23].

### 2.13. Sources of Air Quality, Meteorological, and Respiratory Outpatient Data

Ambient air pollutant data were collected from the Lijia national monitoring station in Chongqing (29.6453° N, 106.5616° E), spanning from 1 January 2021, to 31 December 2024. This station conducted hourly continuous sampling of various pollutants, with PM_2.5_ primarily measured using the beta attenuation method. All air quality data were obtained from the National Urban Air Quality Real-Time Release Platform of the China National Environmental Monitoring Center https://air.cnemc.cn:18007/ (accessed on 5 April 2025). Meteorological data for the same period, including daily average temperature, relative humidity (RH), atmospheric pressure, and wind speed, were sourced from the national centers for environmental information (https://www.ncei.noaa.gov/). All data underwent rigorous quality control procedures.

Respiratory disease outpatient data were collected from the Chongqing Liangjiang New Area People’s Hospital from 2021 to 2024. The raw data were standardized and quality-controlled in accordance with national guidelines and classified according to the International Classification of Diseases, Tenth Revision (ICD-10). The following respiratory diseases were selected as health effect endpoints: COPD (J42–J44), asthma (J45), rhinitis (J30–J31), acute bronchitis (J20), acute upper respiratory infections (J06), and pneumonia (J18). During data processing, non-resident patients of Chongqing were first excluded. Additionally, abnormal fluctuations in respiratory outpatient volumes occurred between December 2022 and March 2023 due to adjustments in domestic COVID-19 prevention policies. Data from this period were omitted from statistical analyses. Finally, the processed data were grouped by sex (male/female), age (<65 years and ≥65 years), and the six categories of respiratory diseases for analysis. Meanwhile, this study protocol has been approved by the Ethics Committee of Chongqing Liangjiang New Area People’s Hospital Approval No. (2026) 02.

### 2.14. Exposure-Lag-Response Relationship

The collated air quality, meteorological, and respiratory outpatient data were used to establish a Generalized Additive Model (GAM) based on Poisson regression using R software (version 4.4.2). Nonlinear confounders were adjusted, including long-term trends, day-of-week, and holidays:

$$\log[E(Y)]\;=\;a\;+\;\beta (PM_{t,l})\;+\;s(\mathrm{temp},k)\;+\;s(\mathrm{RH},k)\;+\;s(P,k)\;+\;\mathrm{ns}(\mathrm{time},\mathrm{df})\;+\;\mathrm{as}.\mathrm{factor}(\mathrm{DOW})\;+\;\mathrm{as}.\mathrm{factor}(\mathrm{holiday})$$

Here, *log[E(Yt)]* is the link function, *α* the intercept, and *β* the regression coefficient for PM_2.5_ (*PMt,l*: lag l days). Smoothing functions-controlled temperature, humidity, and pressure (optimized via QAIC). Time trends were modeled using natural cubic splines (df determined by PACF). Day-of-week (DOW) and holidays were included as dummy variables.

Relative risks (RR) quantified the effect of a 10 μg/m^3^ PM_2.5_ increase on outpatient visits. The maximum lag was set to 7 days. Single-day (lag 0–7) and cumulative (lag 01–07) effects were evaluated.

### 2.15. Statistical Analysis

Experimental measurement data are expressed as mean ± standard error of the mean (Mean ± SEM). All experiments were performed with three biological replicates. Statistical analyses were conducted using SPSS 20 and GraphPad Prism 8 software. Intergroup comparisons were performed using independent-sample *t*-tests. Differences among multiple groups were analyzed by one-way analysis of variance, with the significance level α set at 0.05.

## 3. Results and Discussion

### 3.1. Toxic Effects

This study first evaluated the similarities and differences in the toxic effects of extract of urban and rural PM_2.5_ on respiratory damage. Five aspects were comprehensively assessed: cytotoxicity, ultrastructure, apoptosis, cell cycle, and biomacromolecules.

#### 3.1.1. Cytotoxicity

**Figure 1:**
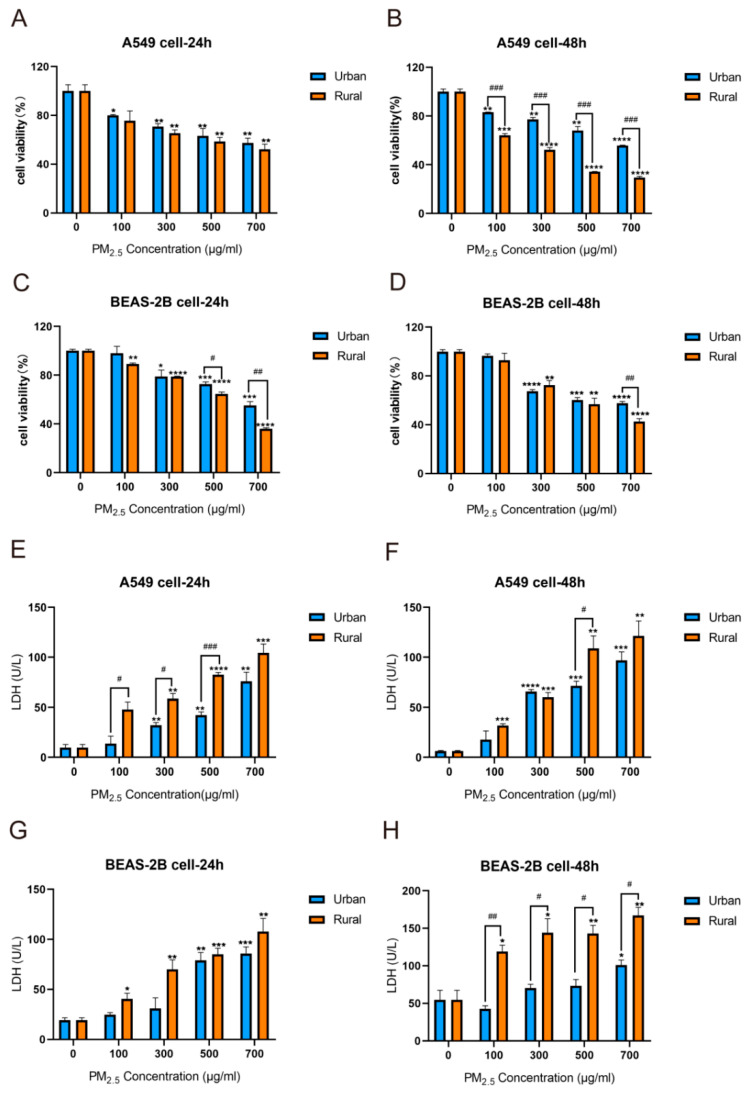
Effects of PM_2.5_ on cell viability inhibition and membrane damage (**A**–**D**): Cell viability; (**E**–**H**): LDH release. (* *p* < 0.05, ** *p* < 0.01, *** *p* < 0.001, **** *p* < 0.0001 vs. control; # *p* < 0.05, ## *p* < 0.01, ### *p* < 0.001 for urban vs. rural PM_2.5_).

CCK8 and LDH assays revealed that both urban and rural PM_2.5_ suppressed the viability and induced cell membrane damage of two lung cell lines (A549 and BEAS-2B) in a concentration- and time-dependent manner (Figure 1). Furthermore, rural PM_2.5_ demonstrated stronger inhibitory effects on viability and greater membrane damage compared to urban PM_2.5_ under the following conditions: for CCK8: A549 (48 h, 100–700 μg/mL), BEAS-2B (24 h, 500–700 μg/mL; 48 h, 700 μg/mL) (Figure 1A–D); for LDH: A549 (24 h, 100–500 μg/mL), A549 (48 h, 500 μg/mL) BEAS-2B (48 h, 100–700 μg/mL) (Figure 1E–H).

Rural PM_2.5_ mainly originates from the incomplete combustion of biomass and coal. Its high content of organic components such as polycyclic aromatic hydrocarbons and quinones results in a significantly higher biotoxicity per unit mass compared to urban PM_2.5_ [24,25]. Furthermore, most people spend the majority of their time indoors, making indoor air quality more critical to human health. Due to the widespread use of solid fuels as the primary energy source in rural areas, indoor PM_2.5_ exposure concentrations are considerably higher in rural than in urban settings [26]. This leads to a potentially greater PM_2.5_ exposure risk for rural populations, which was consistent with our in vitro experiments that rural PM_2.5_ can induce more significant cellular damage than urban PM_2.5_.

#### 3.1.2. Ultrastructure

Having established the cytotoxicity of both urban and rural PM_2.5_, we further investigated their impact on the ultrastructure of A549 and BEAS-2B cells. Findings revealed significant subcellular structural damage from both sources.

**Figure 2:**
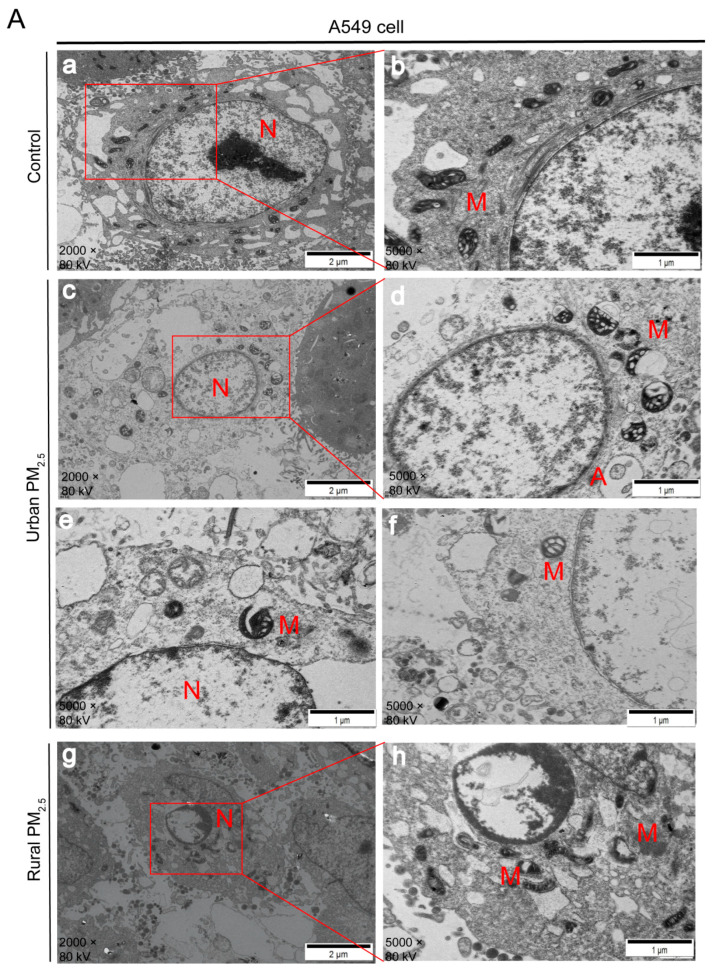

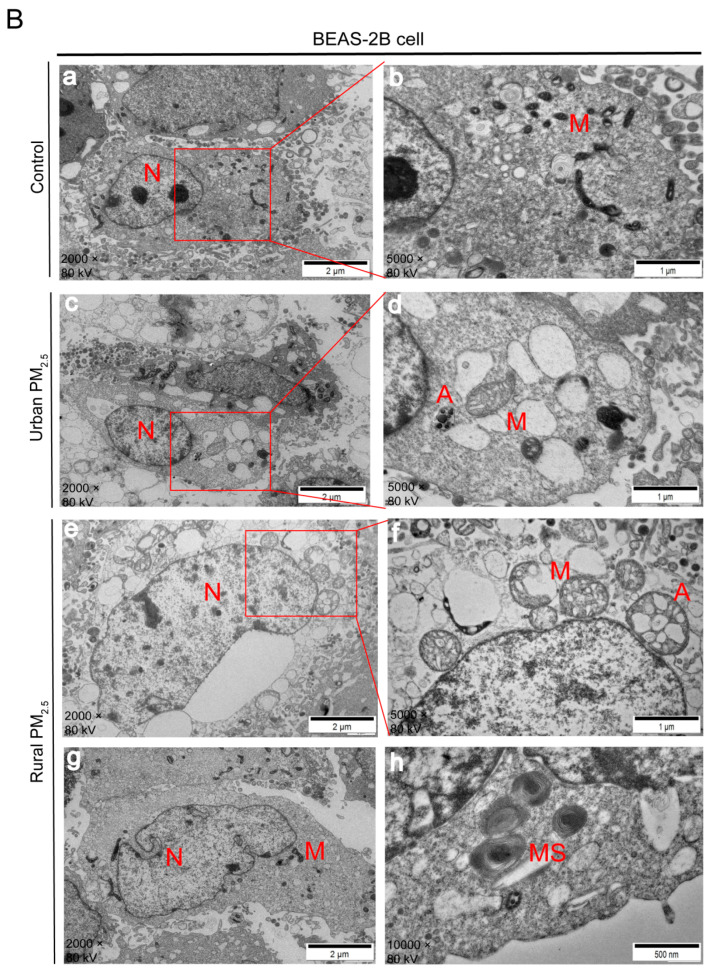
PM_2.5_-induced damage to submicroscopic structures in lung cells (**A**): A549, a, c, g: 2000×, b, d, e, f, h: 5000×; (**B**): BEAS-2B, a, c, e, g: 2000×, b, d, f, h: 5000×; N: Cell nucleus, M: Mitochondria, A: Autophagosome, MS: Myeloid structure.

For A549 cells, urban PM_2.5_ caused chromatin depletion, decreased mitochondrial count, swelling/rupture, cristae reduction, and autophagosomes formation. In contrast, rural PM_2.5_ led to nuclear pyknosis, crescent-shaped chromatin marginalization, and mitochondrial loss with cristae absence (Figure 2A). These results highlight mitochondrial dysfunction from both PM_2.5_ types but distinct nuclear responses. Rural PM_2.5_ induced nuclear pyknosis, while urban PM_2.5_ disrupted chromatin relaxation.

In BEAS-2B cells, urban PM_2.5_ primarily reduced mitochondrial numbers, induced swelling, and generated autophagosomes. However, rural PM_2.5_ caused mitochondrial reduction/swelling, cristae loss, autophagosome formation, invaginated nuclear membranes, and myelin-like structures (Figure 2B). Both PM_2.5_ types impaired mitochondria and activated autophagy in BEAS-2B cells. However, rural PM_2.5_ additionally provoked complex ultrastructural anomalies, such as nuclear invagination and myelin-like structures.

#### 3.1.3. Cell Apoptosis

**Figure 3:**
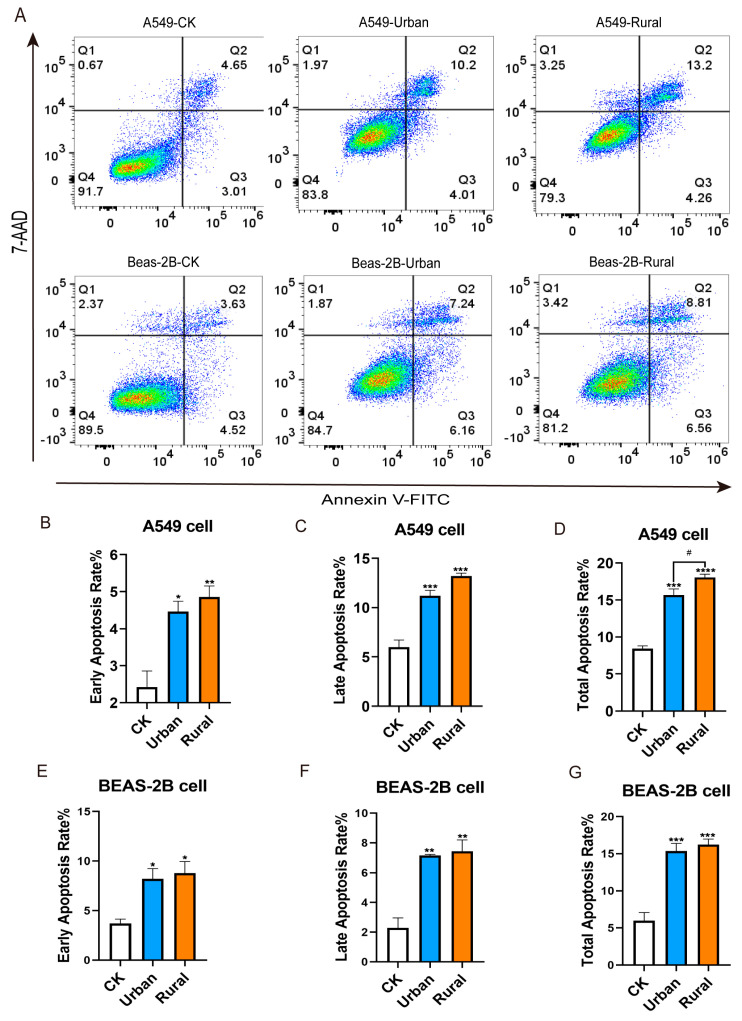
Cells apoptosis induced by PM_2.5_ (**A**) Apoptosis analysis by flow cytometry (**B**,**E**) Early apoptosis rates. (**C**,**F**) Late apoptosis rates. (**D**,**G**) Total apoptosis rates. (* *p* < 0.05, ** *p* < 0.01, *** *p* < 0.001, **** *p* < 0.0001 vs. control; # *p* < 0.05 for urban vs. rural PM_2.5_).

Flow cytometry detection revealed that PM_2.5_ from both urban and rural sources significantly increased the rates of early, late, and total apoptosis in A549 and BEAS-2B cells (Figure 3A–G). This finding is consistent with the documented pro-apoptotic effects of PM_2.5_ [27,28]. Importantly, despite significant apoptosis induction in both cell types, the response profiles differed. For A549 cells, exposure to rural PM_2.5_ resulted in a significantly higher total apoptosis rate compared to urban PM_2.5_ (Figure 3D). However, in BEAS-2B cells, the apoptosis rates at each stage showed no significant difference between urban and rural PM_2.5_ exposure (Figure 3A,E–G). Collectively, these results demonstrate that rural PM_2.5_ exerts a stronger pro-apoptotic effect specifically on A549 cells.

#### 3.1.4. Cell Cycle

**Figure 4:**
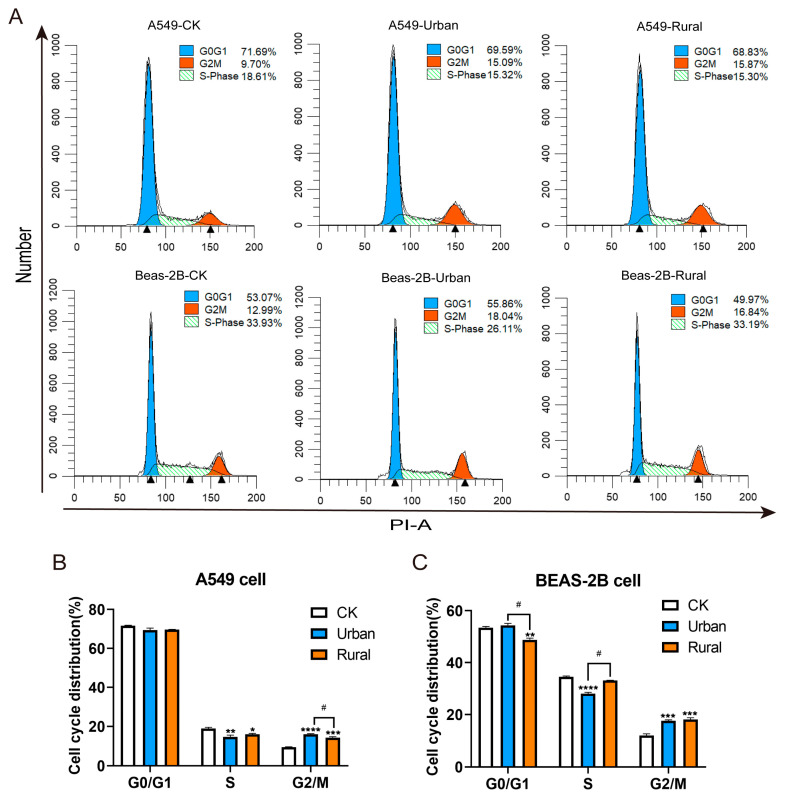
Interference effects of PM_2.5_ on cell cycle (**A**) Cell cycle analysis by flow cytometry (**B**) A549 and (**C**) BEAS-2B. (* *p* < 0.05, ** *p* < 0.01, *** *p* < 0.001, **** *p* < 0.0001 vs. control; # *p* < 0.05 for urban vs. rural PM_2.5_).

Both urban and rural PM_2.5_ exposures decreased the proportion of A549 cells in S phase while increasing the proportion in G2/M phase (Figure 4A,B). This finding is consistent with the known ability of PM_2.5_ to induce G2/M phase arrest in A549 cells [29]. This result indicates potential interference with DNA synthesis (S phase) and DNA damage repair (G2/M phase) as mechanisms disrupting cell cycle progression. Notably, the G2/M phase increase was greater following urban PM_2.5_ exposure compared to rural PM_2.5_, suggesting a stronger disruptive effect on the cell cycle.

In BEAS-2B cells, urban PM_2.5_ similarly caused S phase decrease and G2/M phase increase, aligning with previous reports [30]. However, rural PM_2.5_ exposure in BEAS-2B cells resulted in a decreased in G0/G1 phase proportion and an increased in G2/M phase proportion, leaving S phase unaffected, which was not reported in previous studies. Furthermore, comparing the effects of urban versus rural PM_2.5_ on BEAS-2B cells revealed that urban exposure led to a higher proportion of cells in G0/G1 phase and a lower proportion in S phase (Figure 4A,C). This suggests urban PM_2.5_ is more potent at activating the G0/G1 checkpoint and strongly inhibiting DNA synthesis in BEAS-2B cells.

Consequently, while both urban and rural PM_2.5_ significantly interfere with lung cell cycle progression, their specific modes of action differ depending on cell type and the PM_2.5_ source. These differential cell cycle interference effects may underlie how PM_2.5_ from various sources contributes to respiratory diseases.

#### 3.1.5. Biological Macromolecules

**Figure 5:**
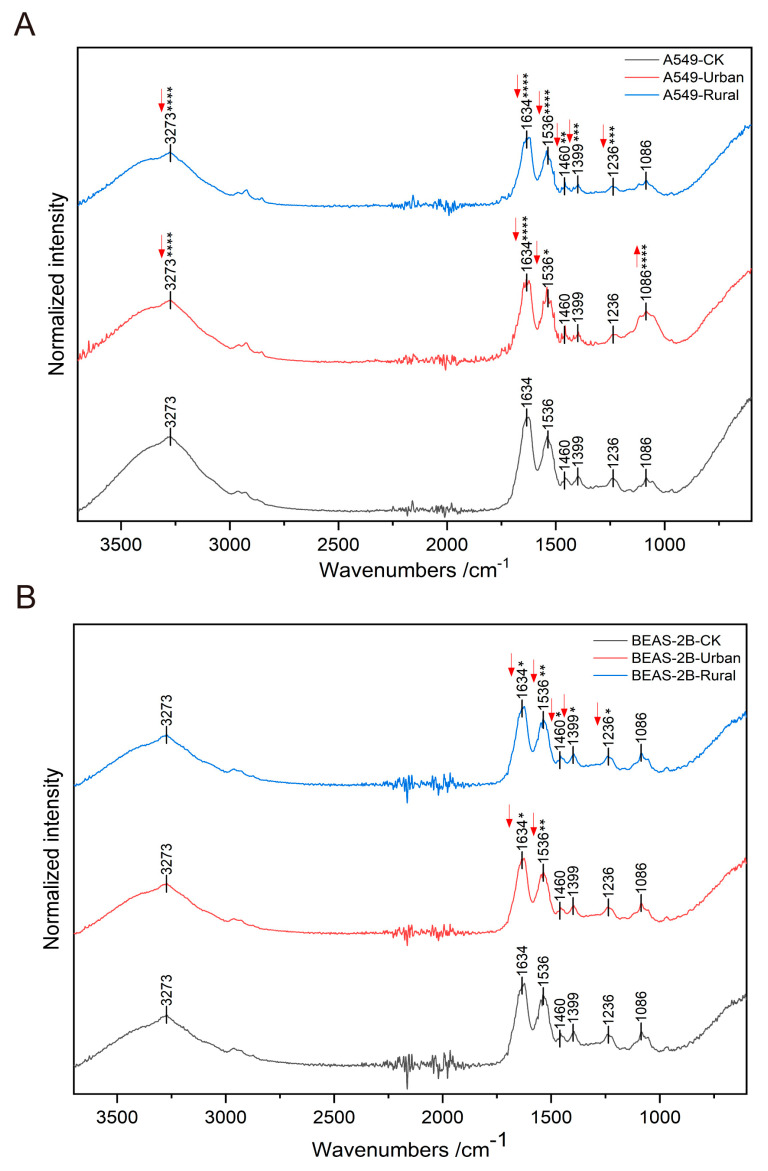
Alterations in biological macromolecules induced by PM_2.5_. (**A**) A549 and (**B**) BEAS-2B cells. * *p* < 0.05, ** *p* < 0.01, *** *p* < 0.001, **** *p* < 0.0001 vs. control. Arrows indicate peak intensity changes (↑ increase; ↓ decrease).

Fourier-transform infrared (FTIR) spectroscopy enables tracking of dynamic changes in biomacromolecules during apoptosis [31]. We further investigated the disruptive effects of urban and rural PM_2.5_ on these biomacromolecules. Figure 5A and Table 1 showed that both PM_2.5_ types induced damage to protein secondary structure in A549 cells. This was evidenced by reduced peaks for Amide A band (3273 cm^−1^), Amide I band (1634 cm^−1^), and Amide II band (1536 cm^−1^). Urban PM_2.5_ specifically intensified the symmetric vibration of phosphate groups (1086 cm^−1^), indicating mild membrane damage through specific disruption of lipid molecular arrangement and interactions. Conversely, rural PM_2.5_ uniquely caused abnormal methyl group vibrations (1460 cm^−1^ and 1399 cm^−1^) and weakened asymmetric phosphate group vibration (1236 cm^−1^), suggesting comprehensive disruption of the phospholipid bilayer and severe membrane damage.

In BEAS-2B cells, urban PM_2.5_ exposure significantly reduced only the peaks at 1634 cm^−1^ and 1536 cm^−1^, signifying interference with protein secondary structure. However, rural PM_2.5_ exposure reduced not only above-mentioned peaks but also those at 1460 cm^−1^, 1399 cm^−1^, and 1236 cm^−1^ (Figure 5B, Table 1), indicating concurrent effects on amide group vibrations, methyl group vibrations, and asymmetric phosphate stretching vibrations. Thus, urban PM_2.5_ primarily impaired protein secondary structure, whereas rural PM_2.5_ additionally compromised the stability of methylation modifications and phosphate groups, inflicting broader structural damage.

In summary, exposure to both urban and rural PM_2.5_ significantly disrupted biomacromolecules in lung cells, with cell-type specific differences. Especially, rural PM_2.5_ induced more extensive biomacromolecular damage across different cell types. Although FTIR is widely used in biology for cellular component analysis [32], its application in PM_2.5_ biotoxicity research remains uncommon. Hence, this study offers a novel methodological approach for investigating PM_2.5_ toxicity.

**Table 1:** Peak band assignment in the FTIR spectrum.

Wavenumbers/(cm^−1^)	Vibration Mode	Major Contributory Ingredients
1086 [33]	νsPO_2_^−^	DNA, RNA, phospholipids
1236 [34,35]	νasPO_2_^−^	DNA, RNA, phospholipids
1399 [36]	δsCH3	Proteins, DNA, RNA
1460 [37]	δasCH3	Proteins, lipids
1536 [38]	ν_C-N_ and δ_N-H_ of amide II	Proteins
1634 [36]	ν_C=O_ of amide I	Proteins
3273 [39]	ν_N-H_ of amide A	Proteins

ν: Stretching vibration; νs: Symmetric stretching motion; νas: Asymmetric stretching motion; δ: Bending vibration; δs: Symmetric bending vibration; δas: Asymmetric bending vibration.

### 3.2. Source Apportionment and Contribution to Toxic Injury from PM_2.5_

We further conducted source apportionment of urban and rural PM_2.5_, aiming to clarify the association between source contributions and toxic injury, thereby enabling a more comprehensive assessment of the health risks posed by PM_2.5_ exposure in both areas.

**Figure 6:**
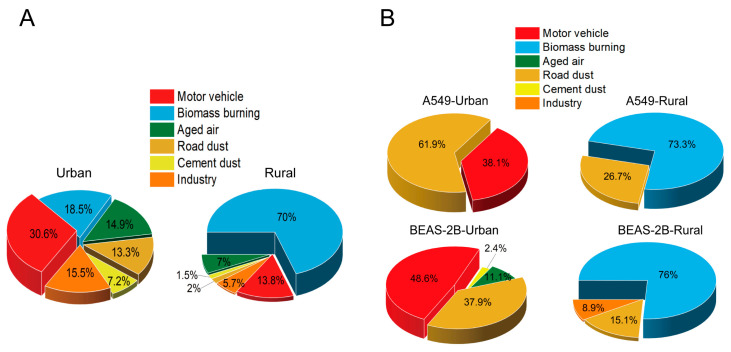
Sources of PM_2.5_ and their toxicological effects (**A**): PM_2.5_ sources; (**B**): contribution of PM_2.5_ Sources and Their Toxic Effects (Note: All data was within the criteria of R squared > 0.8 and *p*-value < 0.05).

Significant differences were observed in pollution source profiles between urban and rural PM_2.5_ (Figure 6A). Urban PM_2.5_ exhibited a mixed pollution pattern, with contributions from motor vehicles (30.6%), biomass burning (18.5%), and industrial emissions (15.5%). Conversely, rural PM_2.5_ was dominated by a single source: biomass burning (70%). Furthermore, multiple linear regression analysis revealed distinct urban-rural differences in the sources responsible for PM_2.5_-induced cytotoxicity (Figure 6B). In urban settings, road dust and motor vehicle emissions emerged as the top contributors to cytotoxicity in both A549 and BEAS-2B cell lines. In rural areas, biomass burning was the dominant cytotoxic source. Notably, road dust was identified as a key contributor to lung cell toxicity induced by PM_2.5_ in both environments.

### 3.3. Mechanistic Insights into PM_2.5_-Induced Toxic Damage from Dual-Omics Analysis

We subsequently conducted transcriptomic and proteomic sequencing to systematically reveal the common and distinct molecular regulatory mechanisms underlying lung cell damage induced by urban and rural PM_2.5_.

#### 3.3.1. Dual-Omics: Key Molecules

The differentially expressed genes (DEGs) and proteins (DEPs) induced by urban and rural PM_2.5_ are presented in Table S1A–H. Subsequently, the STRING database and CytoHubba 3.8.2software were employed to identify both shared and unique key molecular targets of urban and rural PM_2.5_ (Table S2A–E).

**Figure 7:**
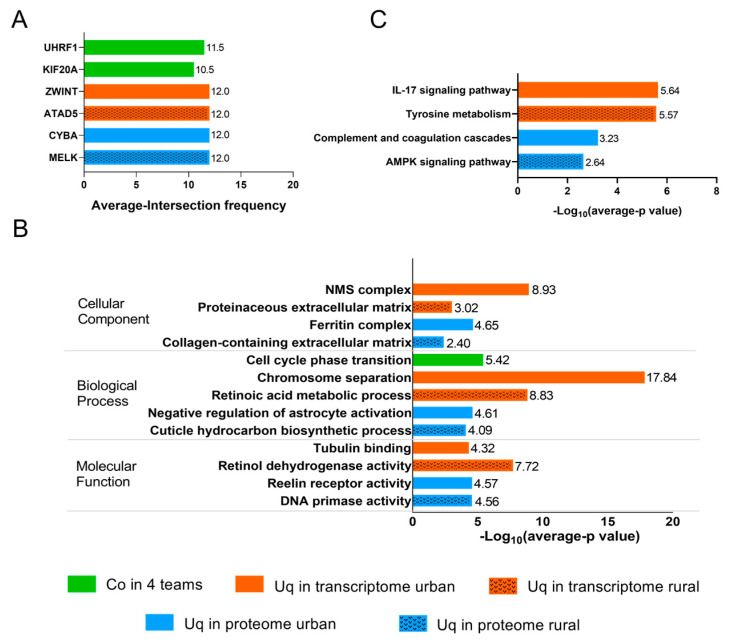
Key molecules, GO terms, and pathways regulated by PM_2.5_ (top 1) (**A**) key molecules; (**B**) key GO terms; (**C**) key KEGG pathways.

Joint analysis revealed that urban and rural PM_2.5_ simultaneously target *UHRF1* and *KIF20A* at both mRNA and protein levels (Figure 7A). Notably, *KIF20A*, a member of the kinesin family, plays a critical role in the G2/M phase of the cell cycle, thereby regulating cell division [40,41]. It is closely associated with respiratory diseases. However, its link to PM_2.5_-induced respiratory damage has not been established. Flow cytometry results in this study showed that both urban and rural PM_2.5_ exposure induce G2/M phase arrest, which was highly consistent with the known functions of *KIF20A*, suggesting its potential pivotal role in PM_2.5_-induced lung cell damage. Therefore, subsequent mechanistic studies will focus on elucidating the role of *KIF20A* in this process.

Analysis of specific molecular targets indicated that urban and rural PM_2.5_ predominantly disrupted *ZWINT* and *ATAD5* only at the mRNA level, respectively. *ZWINT* is a core mitotic regulator essential for centromere formation and spindle checkpoint activity [42]. It is linked to lung cancer and pulmonary fibrosis [43,44]. *ATAD5* participates in PCNA (proliferating cell nuclear antigen) deubiquitylation, thereby interfering with DNA damage repair. It may contribute to respiratory diseases (e.g., lung adenocarcinoma and pulmonary valve stenosis) by inducing DNA mutations [45,46].

At the protein level only, the primary specific targets were CYBA (urban) and MELK (rural). CYBA is involved in oxidative stress [47] and associated with lung abscess, asthma, and pneumoconiosis [48,49,50], while MELK regulates cell cycle progression [51,52] and is implicated in lung tumors, nasopharyngeal carcinoma, and pulmonary fibrosis [53,54,55].

These specific targets (*ZWINT*, *ATAD5*, CYBA and MELK) have not been previously reported in PM_2.5_-related studies. Therefore, this study is the first to reveal their potential specific roles in health risks associated with urban and rural PM_2.5_ exposure.

#### 3.3.2. Dual-Omics: Key GO Terms

The biological functions regulated by urban and rural PM_2.5_ at the mRNA and protein levels are shown in Table S3A–E. Subsequently, the top-ranked shared and unique biological functions were analyzed from three levels: cellular component (CC), biological process (BP), and molecular function (MF) (Figure 7B).

In CC, no shared targets were identified between urban and rural PM_2.5_. At the mRNA level, the most specific CCs regulated by urban and rural PM_2.5_ were the NMS complex and proteinaceous extracellular matrix, respectively. The former primarily participates in ribosomal RNA processing and protein synthesis, contributing to lung tumorigenesis [56]. The latter maintains cellular structural stability and is associated with asthma and COPD [57]. At the protein level, the most prominent urban-specific CC was the ferritin complex. Its dysfunction induces oxidative stress [58] and promotes pulmonary fibrosis [59]. In contrast, rural PM_2.5_ specifically targeted the collagen-containing extracellular matrix, whose abnormal deposition and remodeling are key drivers of fibrosis [60], closely linked to lung tumors and COPD [61,62]. Thus, dual-omics analysis further highlights the stark differences in CCs regulated by urban and rural PM_2.5_ at either the mRNA or protein level.

In BP, cell cycle phase transition was the most prominent process co-regulated by urban and rural PM_2.5_ at both mRNA and protein levels. As a core regulatory mechanism of the mitotic cell cycle, it governs phase transitions to ensure proper mitosis [63]. It is implicated in acute lung injury [64]. This suggests that urban and rural PM_2.5_ may disrupt the cell cycle at both mRNA and protein levels for triggering respiratory diseases, which was consistent with flow cytometry results in toxicity experiment. At the transcriptomic level, the most significant urban and rural-specific processes were chromosome segregation and retinoic acid metabolic process, respectively. Chromosome segregation, a pivotal step in cell division, is linked to lung tumorigenesis [65,66], while retinoic acid metabolic process regulates retinoic acid synthesis, which is critical for bronchial and lung development [67,68]. At the proteomic level, the most prominent urban-specific process was negative regulation of astrocyte process. This process induces cellular inflammation by modulating central nervous system activation and plays a key role in lung tumorigenesis [69]. Rural PM_2.5_ uniquely regulated cuticular hydrocarbon biosynthetic process. This process primarily involved in arthropod cuticle formation but has reported potential roles in lung tumorigenesis [60,70,71]. These findings underscore the highly specific BPs through which urban and rural PM_2.5_ drive respiratory diseases.

In MF, no shared targets were identified between urban and rural PM_2.5_ in dual-omics analysis. At the transcriptomic level, urban PM_2.5_ predominantly regulated tubulin binding. This function participates in cell division and chromosome segregation, potentially contributing to lung tumorigenesis [72,73]. Rural PM_2.5_ specifically targeted retinol dehydrogenase activity, which modulates retinoic acid biosynthesis and is associated with asthma and COPD [74,75,76]. At the proteomic level, urban PM_2.5_ uniquely regulated reelin receptor activity, though no direct link to respiratory diseases has been established. Rural PM_2.5_ uniquely influenced DNA primase activity, a critical step in DNA replication implicated in COPD [70]. Collectively, these MFs delineate the divergent mechanisms by which urban and rural PM_2.5_ promote respiratory diseases.

#### 3.3.3. Dual-Omics: Key Signaling Pathways

The key signaling pathways regulated by urban and rural PM_2.5_ at the mRNA and protein levels are shown in Table S4A–D. No shared pathways were identified between urban and rural PM_2.5_ (Figure 7C).

At the mRNA level, the most significantly specific pathways for urban and rural PM_2.5_ were interleukin-17 (IL-17) signaling and tyrosine metabolism, respectively. Studies have confirmed that IL-17 is involved in the regulation of asthma and COPD [77]. Tyrosine metabolism participates in the pathogenesis of lung cancer, pulmonary fibrosis, and asthma [78,79]. Therefore, at the mRNA level, urban and rural PM_2.5_ may contribute to respiratory diseases development through IL-17-mediated immune dysregulation and abnormal tyrosine metabolism, respectively.

At the protein level, urban PM_2.5_ primarily and specially affected the complement and coagulation cascades. Interfering with complement activation can destroy the viral clearance function of immune cells, increasing the risk of upper respiratory tract infections [80]. Rural PM_2.5_ specially targeted the AMPK signaling pathway, disrupting cellular energy metabolism [81], thereby exacerbating pulmonary fibrosis and lung injury [82]. Consequently, at the protein level, urban PM_2.5_ specially disrupts cellular immunity via inflammatory pathways, whereas rural PM_2.5_ impairs energy metabolism by inhibiting the AMPK pathway, leading to respiratory diseases.

Therefore, urban and rural PM_2.5_ drive respiratory diseases through the regulation of specific rather than shared signaling pathways.

### 3.4. Functional Validation of KIF20A in PM_2.5_-Induced Toxicity Damage

The dual-omics analysis results indicated that KIF20A is a common key molecule in lung cell damage induced by both urban and rural PM_2.5_. Therefore, this study further validated the function of KIF20A.

#### 3.4.1. PM_2.5_ Exposure Inhibits KIF20A Protein Expression

Dual-omics data revealed that both urban and rural PM_2.5_ significantly reduced the mRNA and protein expression levels of KIF20A. Western blot confirmed that exposure to urban and rural PM_2.5_ significantly suppressed KIF20A protein expression, validating the dual-omics results (Figure S1A,C). Subsequently, a KIF20A overexpression vector was constructed and verified by Western blot (Figure S1B,D).

#### 3.4.2. Effect of KIF20A Overexpression on PM_2.5_-Induced Toxic Damage

**Figure 8:**
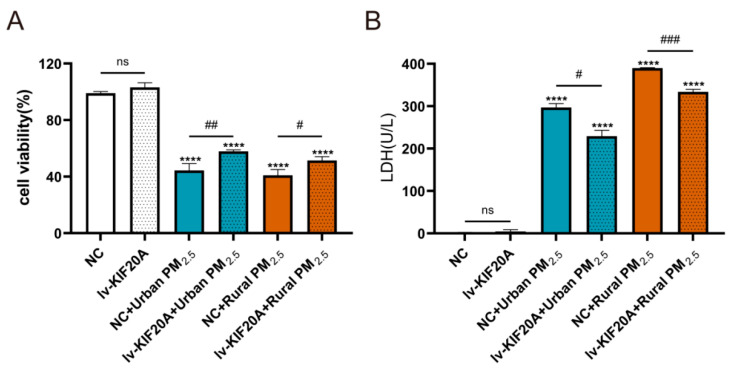
Role of KIF20A in cytotoxicity induced by urban and rural PM_2.5_ (**A**) Cell viability and (**B**) LDH release. (**** *p* < 0.0001 vs. control; ns *p* > 0.05, # *p* < 0.05, ## *p* < 0.01, ### *p* < 0.001 for KIF20A overexpression groups).

Rescue experiments demonstrated that KIF20A overexpression alleviated the reduction in cell viability and the elevation in LDH release induced by urban and rural PM_2.5_ (Figure 8A,B). However, FTIR spectroscopy analysis indicated that KIF20A overexpression did not ameliorate the molecular structural disturbances caused by urban and rural PM_2.5_ (Figure S2).

**Figure 9:**
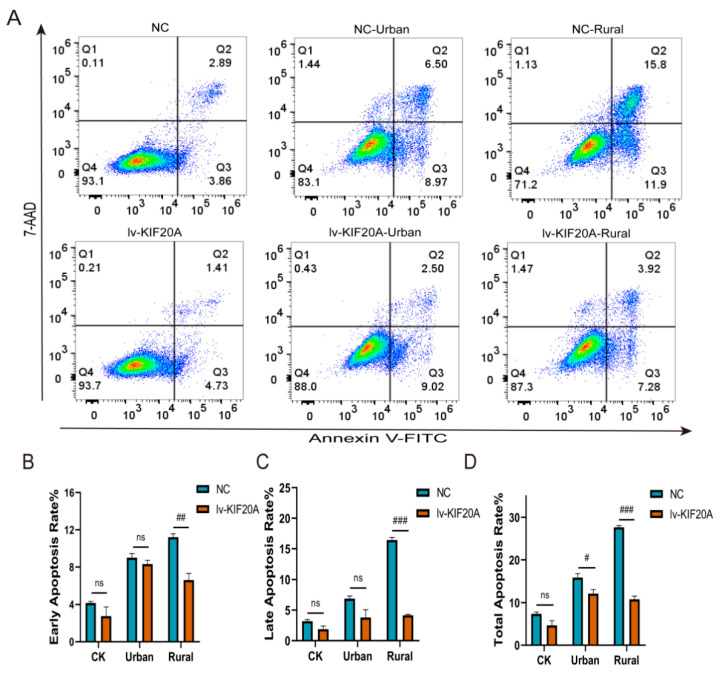
Role of KIF20A in PM_2.5_-induced cell apoptosis (**A**) apoptosis analysis, (**B**) early apoptosis rates, (**C**) late apoptosis rates, (**D**) total apoptosis rates. (ns *p* > 0.05, # *p* < 0.05, ## *p* < 0.01, ### *p* < 0.001 for KIF20A overexpression groups).

As a critical motor protein essential for chromosomal segregation and cytokinesis during mitosis, KIF20A primarily functions in the G2/M phase [41]. Consequently, KIF20A likely participates in PM_2.5_-induced apoptotic responses and cell cycle perturbations in both urban and rural samples. Rescue assays demonstrated that upregulating KIF20A markedly reduced the total apoptosis rate triggered by urban PM_2.5_. Furthermore, it decreased the early, late, and total apoptosis rates caused by rural PM_2.5_ (Figure 9A–D).

**Figure 10:**
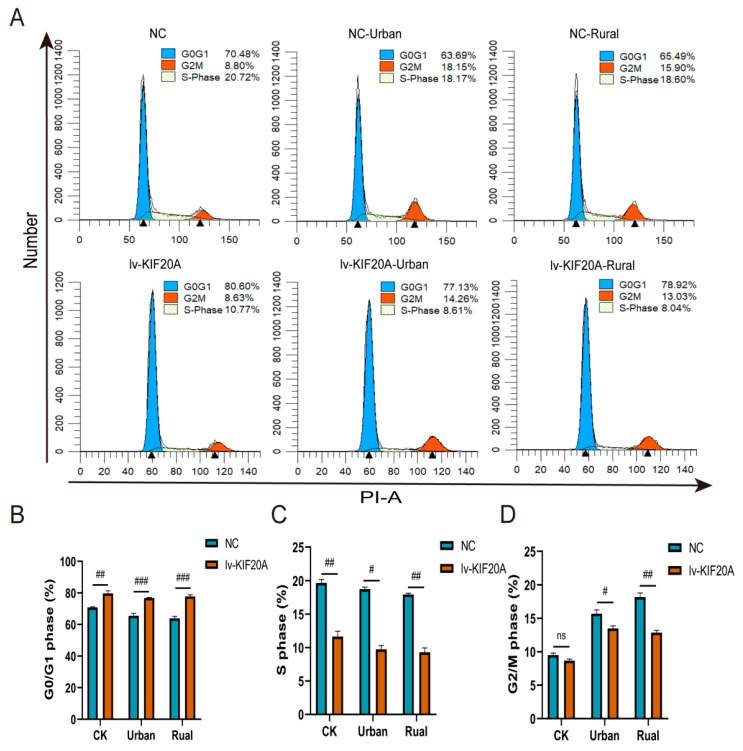
Role of KIF20A in cell cycle dysregulation induced by PM_2.5_ (**A**) Cell cycle analysis. (**B**) G0/G1, (**C**) S, and (**D**) G2/M phase distributions. (ns *p* > 0.05, # *p* < 0.05, ## *p* < 0.01, ### *p* < 0.001 for KIF20A overexpression groups).

In cell cycle rescue experiments (Figure 10A–D), KIF20A + PM_2.5_ exposure resulted in a higher percentage of cells in G0/G1 phase. Concurrently, a decline in S phase and G2/M phase populations was observed compared to the PM_2.5_ group. These findings suggest that while KIF20A overexpression counteracts PM_2.5_-induced G2/M phase arrest, it does not prevent the reduction in S phase cells, which aligns with prior reports on KIF20A’s primary role in G2/M regulation [41]. The concomitant rise in G0/G1 phase cells implies that KIF20A may promote M phase completion, enabling more cells to progress into G0/G1.

In conclusion, KIF20A overexpression mitigates apoptotic effects and alleviates G2/M phase arrest associated with urban and rural PM_2.5_ exposure.

### 3.5. Lagged Exposure-Response Relationship Between Urban PM_2.5_ and Respiratory Outpatient Visits

After clarifying the common and specific effects of urban and rural PM_2.5_ on lung cells, this study aims to explore the correlation between PM_2.5_ exposure and respiratory diseases in both settings using epidemiological data. However, rural areas face challenges including underdeveloped monitoring systems, scattered data sources, and limited availability of respiratory outpatient record. Systematic data collection from rural sampling sites remains difficult. Therefore, considering data reliability and continuity, respiratory outpatient data from a class A tertiary hospital near the urban PM_2.5_ sampling site (2021–2024) were selected for the urban PM_2.5_ exposure group analysis. This dataset possesses relatively complete time-series characteristics, providing a reliable basis for analyzing the association between urban PM_2.5_ exposure and respiratory diseases, thereby validating toxicological experimental findings.

#### 3.5.1. Descriptive Statistics of Urban Respiratory Outpatient Visits

From 2021 to 2024, a total of 83,181 respiratory outpatient visits were recorded in the hospital, demonstrating a consistent annual increase (2021 < 2022 < 2023 < 2024). Females accounted for 44,602 visits and males for 38,579 visits. The majority of visits (61,367) were from individuals under 65 years of age, whereas those aged 65 years and older contributed 21,814 visits. These findings indicate that females outnumbered males in outpatient visits, with young and middle-aged adults being the predominant demographic, and the elderly constituting a relatively smaller proportion.

With respect to disease distribution, the most frequent diagnoses, in descending order of total visits, were acute bronchitis (26,449), upper respiratory tract infection (18,531), asthma (9353), rhinitis (3731), pneumonia (3698), and chronic obstructive pulmonary disease (1749). Acute bronchitis and upper respiratory tract infection together represented 70.82% of the total visits for these six conditions, with maximum daily visits of 97 and 99, respectively—markedly higher than the other diseases—suggesting that these two conditions impose the major disease burden on respiratory outpatient clinics in this hospital. Details of respiratory outpatient data are presented in Table S5.

#### 3.5.2. Population

**Figure 11:**
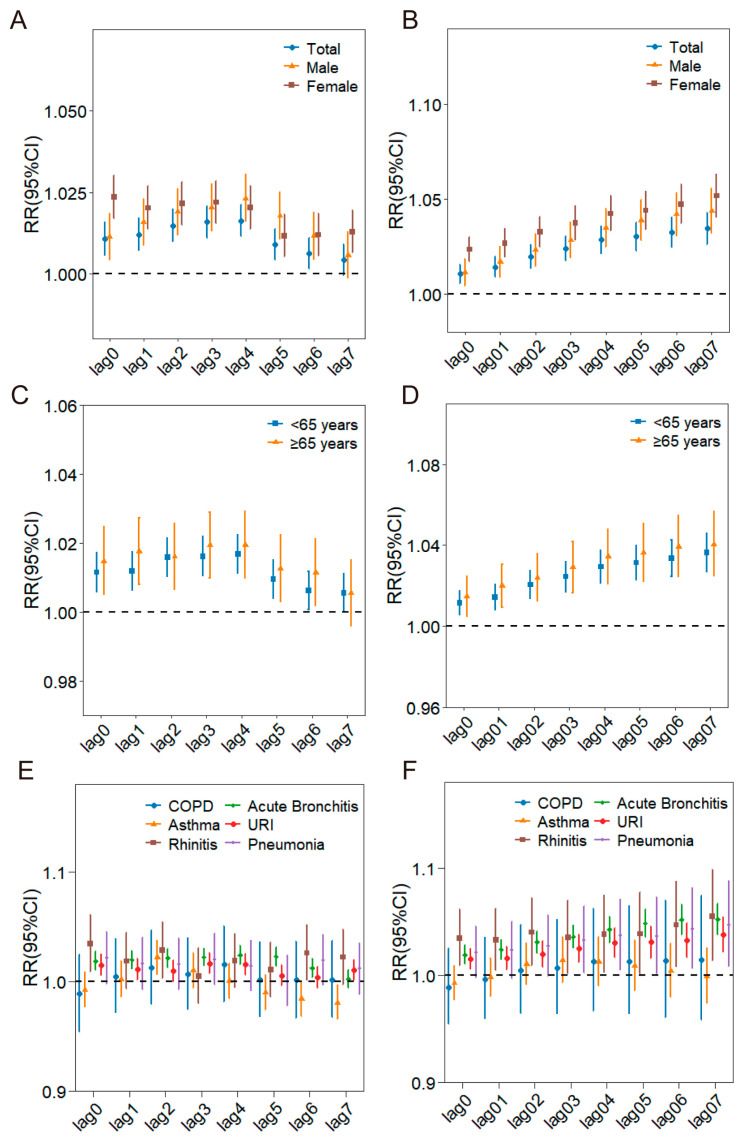
Single-day and cumulative lag risk of PM_2.5_ on urban respiratory diseases outpatient visits (**A**,**C**,**E**): Single-day lag effects (lag 0–7); (**B**,**D**,**F**): Cumulative lag effects (lag 0–07).

Research demonstrates significant lagged impacts of PM_2.5_ concentration increases (per 10 μg/m^3^) on respiratory outpatient visits (Figure 11A). Single-day exposure effects for the total population show that risk rises immediately on the exposure day (lag 0, RR = 1.012), peaks at lag 4 (RR = 1.016), and becomes non-significant at lag 7. Gender differences are notable: females exhibit greater and more immediate sensitivity, reaching peak risk on the exposure day lag 0 (RR = 1.024). In contrast, males show a delayed response, with risk peaking at lag 4 (RR = 1.023). Although no significant difference was observed between genders (*p* > 0.05), the values for males were generally lower than those for females in most lag periods. This suggests females experience more acute reactions versus delayed responses in males.

Regarding cumulative exposure effects, the relative risk (RR) increases lag duration for all groups, reaching maximum at lag 07 (Figure 11B). The relative risk (RR) was 1.052 for females and 1.044 for males. Although the difference between the sexes did not reach statistical significance, the data still indicated a trend of higher cumulative health risks associated with PM_2.5_ exposure among females.

The findings indicate that females exhibit a more rapid outpatient response to single-day PM_2.5_ exposure, whereas males show a relatively delayed response pattern. Although there are gender differences in the timing of response, the ultimate effect intensity between males and females is not significantly different. The results of this study was consistent with the observations in short-term exposure studies that females may face higher risks [83,84]. This discrepancy may stem from differences in exposure patterns or population characteristics examined in this study, which prevented observation of a significant trend and led only to the conclusion that females are more sensitive to health risks than males.

#### 3.5.3. Age

Single-day lag effects of PM_2.5_ across age groups are shown in Figure 11C. Although the difference between age groups was not statistically significant (*p* > 0.05), during lag 0–lag 6, RR values for individuals aged ≥ 65 years consistently exceeded those of the <65 group, with both groups reaching peak risk at lag 4. This suggests that older adults may face higher acute health risks from PM_2.5_ exposure.

Regarding cumulative lag effects (Figure 11D), the cumulative risk for the ≥65 group also showed a consistently higher trend compared to the <65 group. The RR values for both groups increased with longer lag periods, peaking at lag 07 (younger group RR = 1.036; older group RR = 1.041). Thus, older adults may bear a greater cumulative health burdens from PM_2.5_.

The study showed that although individuals aged ≥ 65 years exhibited a more pronounced trend in health risks compared to younger adults, the differences between groups were not significant. Be consistent with previous studies [85], an age trend was present despite the lack of statistical significance under PM_2.5_ exposure. These findings may be related to factors such as the exposure levels or sample size of the study population. Future studies should further explore potential differences between age groups under a wider range of exposure conditions.

#### 3.5.4. Diseases

Single-day lag effect analysis revealed differential risks of PM_2.5_ exposure on various respiratory diseases (Figure 11E). Specifically, acute bronchitis showed sustained risks from lag 0 to lag 6, peaking at lag 4 (RR = 1.024). Upper respiratory infections demonstrated risks during lag 0–lag 4 and lag 7, peaking at lag 3 (RR = 1.017). Rhinitis exhibited risks at lag 0, 2, and 6, with maximum effect at lag 0 (RR = 1.034). Asthma displayed transient risk elevation only at lag 2 (RR = 1.002), while pneumonia and COPD showed no significant risks. Despite the lack of statistical significance between diseases (*p* > 0.05), ordering them by the highest single-day RR showed that: rhinitis (lag 0, RR = 1.034) > acute bronchitis (lag 4, RR = 1.024) > upper respiratory infection (lag 3, RR = 1.017) > asthma (lag 2, RR = 1.002). These findings indicate that PM_2.5_ may exerts the most prominent acute effect on rhinitis with immediate peak risk upon exposure. The risks of acute bronchitis and upper respiratory infections peaked several days after exposure, while asthma manifested only transient risk.

In cumulative exposure analysis (Figure 11F), RR values for acute bronchitis, upper respiratory infections, and rhinitis progressively increased from lag 0 onward. Pneumonia demonstrated delayed cumulative risk starting from lag 03, whereas COPD and asthma showed no significant cumulative effects. By lag 07, the RR trend is as follows: rhinitis (1.055) > acute bronchitis (1.052) > pneumonia (1.047) > upper respiratory infection (1.037), suggesting prolonged PM_2.5_ exposure significantly increases cumulative risks for these diseases, with rhinitis being most prominent.

These results reveal disease-specific variations in urban PM_2.5_ health effects. Rhinitis, acute bronchitis, and upper respiratory infections exhibited both acute and cumulative risks, whereas pneumonia manifested only delayed cumulative effects. PM_2.5_ may increase rhinitis risk by disrupting nasal epithelial cell metabolism, impairing epithelial barrier integrity, and altering mucociliary clearance [86]. Simultaneously, PM_2.5_-bound metallic components could act as inflammatory triggers for pneumonia and upper respiratory infections [87]. In this study, we did not observe significant differences among the various diseases, which may be attributed to the lack of clear distinction between warm and cold seasons, as well as interference from other pollutants.

### 3.6. The Association Among Toxicity, Pathogenesis and Epidemiology

We confirmed that both urban and rural PM_2.5_ can induce cellular damage, mitochondrial damage, cellular apoptosis, and G2/M phase arrest; these are foundational events in the pathogenesis of respiratory diseases. Studies demonstrate that, PM_2.5_ can directly compromise the integrity of airway epithelial cell membranes through physical damage mechanisms, causing conformational disorganization and functional impairment of phospholipid bilayer structures [88]. The resultant breach in the airway epithelial barrier creates pathways for pathogen invasion, thereby predisposing to upper respiratory tract disorders such as rhinitis [89,90]. Concurrently, the disruption of epithelial barrier architecture further enhances permeability in alveolar and bronchial epithelial cells, facilitating massive inflammatory cell infiltration. This process ultimately establishes a pro-pathological microenvironment conducive to the development of acute bronchitis and pneumonia [91,92].

PM_2.5_ initiates mitochondrial injury, which can trigger deficits in ATP production and aberrant cellular energy metabolism, ultimately manifesting as weakened airway clearance mechanisms, heightened risk of pneumonia [93]. Apoptosis of airway epithelial cells contributes to airway hyperresponsiveness by impairing barrier function and exposing nerve endings, a process widely recognized as a key pathological basis for asthma pathogenesis [94,95]. Notably, PM_2.5_ caused cell cycle arrest (e.g., G2/M phase). This process not only impairs epithelial repair capacity but also promotes cellular senescence-associated secretory phenotype, which establish a chronic inflammatory microenvironment of asthma [89,96]. Therefore, toxicological phenotypes confirmed in this study establish pathological foundations for respiratory diseases involved in epidemiological associations.

We also identified KIF20A as a target of both PM_2.5_. According to the Comparative Toxicogenomic Database, this gene is associated with multiple respiratory diseases, such as asthma, pneumonia, respiratory tract infections, and rhinitis. These findings are consistent with epidemiological evidence linking urban PM_2.5_ exposure to the aforementioned diseases, thereby strengthening the evidence for a critical role of KIF20A in PM_2.5_-induced respiratory disorders.

## 4. Limitations

The limitations of this study include the following. First, the study relied on cellular models without in vivo animal validation. Second, PM_2.5_ samples were collected only during the winter months. Third, the study was based on urban outpatient data without parallel rural population data, and did not rigorously distinguish between warm and cold seasons. Furthermore, the use of broad respiratory disease categories may have masked the specific exposure-response dynamics for conditions such as allergic rhinitis or chronic bronchitis.

## 5. Conclusions

This study systematically compared the health risks associated with PM_2.5_ exposure in urban and rural areas of the Chengdu-Chongqing economic circle, revealing significant urban-rural differences. The results indicated that PM_2.5_ from both areas significantly induced cytotoxic damage, with rural PM_2.5_ exhibiting stronger toxic effects. Source apportionment of toxicity showed that the cytotoxicity of urban PM_2.5_ primarily originated from road dust and vehicle emissions, while biomass burning was the dominant source of toxicity for rural PM_2.5_. At the molecular level, KIF20A was identified for the first time as a common key mediator of lung cell damage induced by PM_2.5_ from both areas. Epidemiological analysis revealed that females and ≥65 years old exhibit relatively high sensitivity to urban PM_2.5_ exposure trends. Among various diseases, the exposure trend of rhinitis is the most pronounced. This study clarifies the common and unique geographic patterns of PM_2.5_-induced respiratory risk in urban and rural settings by delineating toxic effects, source risk, molecular pathways, population susceptibility, and disease vulnerabilities, thereby offering a scientific foundation for tailored air quality management strategies.

## Data Availability

The original contributions presented in this study are included in the article/Supplementary Materials. Further inquiries can be directed to the corresponding authors.

## Supplementary Materials

The following supporting information can be downloaded at: https://www.mdpi.com/article/10.3390/toxics14060531/s1, KIF20A protein expression in A549 cells (Figure S1); KIF20A mediates urban and rural PM_2.5_-induced disruption of biomolecular structures (Figure S2); DEGs and DEPs (Table S1), key molecules (Table S2), GO terms (Table S3), KEGG pathways (Table S4), Daily Outpatient Visits Distribution of Urban Respiratory Diseases (Table S5).
