# Supplementary material for: Common and Unique Respiratory Health Risk Induced by Urban-Rural PM2.5 in the Chengdu-Chongqing Economic Circle
Source: Toxics. 2026 Jun 20;14(6):531. doi: 10.3390/toxics14060531 (PMC13307873; doi:10.3390/toxics14060531)
Supplement: Supplementary file 1 [file toxics-14-00531-s001.zip › Supplementary figures.pdf]

Supplementary figures

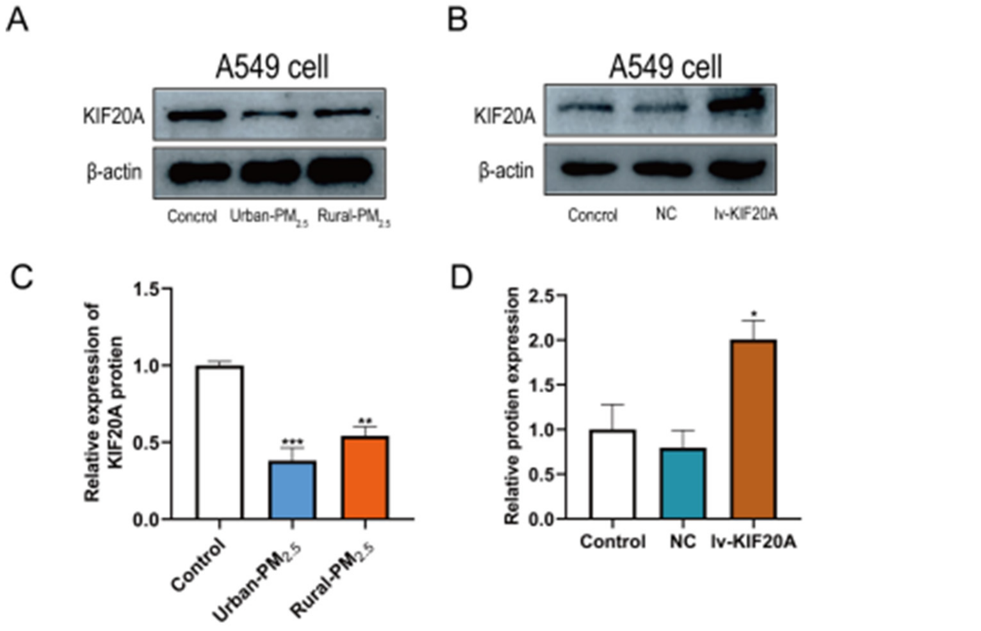

Fig. S1 Cellular KIF20A protein expression levels and overexpression vector construction under urban and rural $PM_{2.5}$ exposure.

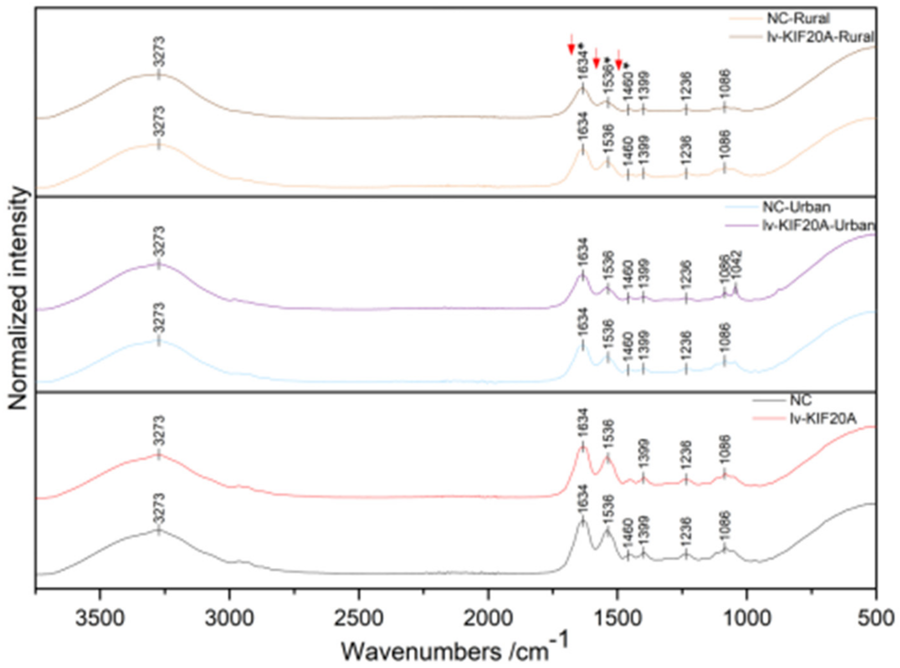

Fig. S2 Role of KIF20A in the perturbation of macromolecular structures by urban and rural $PM_{2.5}$.
