# Supplementary material for: Common and Unique Respiratory Health Risk Induced by Urban-Rural PM2.5 in the Chengdu-Chongqing Economic Circle
Source: Toxics. 2026 Jun 20;14(6):531. doi: 10.3390/toxics14060531 (PMC13307873; doi:10.3390/toxics14060531)
Supplement: Supplementary file 1 [file toxics-14-00531-s001.zip › Supplementary tables S5.pdf]

Supplementary Tables S5. Daily Outpatient Visits Distribution of Urban Respiratory Diseases

Disease Classification	Total	Mean ± SD	Min	Max	Percentile			
					25	50	75	95
Total Outpatient Visits	83181	81±45	7	244	48	67	104	170
2021 year	15392	45±10	7	75	37	44	52	61
2022 year	14685	49±14	10	85	40	49	58	74
2023 year	19770	84±33	32	153	55	76	115	141
2024 year	33334	110±49	7	244	73	93	151	211
Male	38579	37±19	2	115	23	31	46	78
Female	44602	45±27	3	133	26	37	59	98
Age < 65 Years	61367	63±38	3	205	35	49	83	140
Age ≥ 65 Years	21814	20±9	1	51	13	19	26	39
Chronic Obstructive Pulmonary Disease(COPD)	1749	3±2	1	12	2	3	5	8
Asthma	9353	10±7	1	49	6	9	13	22
Rhinitis	3731	7±6	1	40	3	5	9	20
Acute Bronchitis	26449	30±20	1	97	15	23	40	73
Upper Respiratory Tract Infection(URTI)	18531	27±22	1	99	11	18	43	70
Pneumonia	3698	5±3	1	16	3	4	6	11
